# Acute and chronic cardiometabolic responses induced by resistance training with blood flow restriction in HIV patients

**DOI:** 10.1038/s41598-022-19857-3

**Published:** 2022-10-10

**Authors:** Thiago Cândido Alves, Pedro Pugliesi Abdalla, Lucimere Bohn, Leonardo Santos Lopes Da Silva, André Pereira dos Santos, Márcio Fernando Tasinafo Júnior, Ana Cláudia Rossini Venturini, Jorge Mota, Dalmo Roberto Lopes Machado

**Affiliations:** 1 Universidade de São Paulo, Avenue of Bandeirantes no 3900, University Campus-Monte Alegre, Ribeirão Preto, SP 14040-902 Brazil; 2 Universidade do Porto, Porto, Portugal; 3 Universidade Lusófona do Porto, Porto, Portugal

**Keywords:** Cardiology, Diseases, Health care

## Abstract

Resistance training with blood flow restriction (RTBFR) allows physically impaired people living with HIV (PWH) to exercise at lower intensities than traditional resistance training (TRT). But the acute and chronic cardiac and metabolic responses of PWH following an RTBFR protocol are unknown. The objective was to compare the safety of acute and chronic effects on hemodynamic and lipid profiles between TRT or RTBFR in PWH. In this randomized control trial, 14 PWH were allocated in RTBFR (G_RTBFR_; n = 7) or TRT (G_TRT;_ n = 7). Both resistance training protocols had 36 sessions (12 weeks, three times per week). Protocol intensity was 30% (G_RTBFR_) and 80% (G_TRT_). Hemodynamic (heart rate, blood pressure) and lipid profile were acutely (rest and post exercise 7th, 22nd, and 35th sessions) and chronically (pre and post-program) recorded. General linear models were applied to determine group * time interaction. In the comparisons between groups, the resistance training program showed acute adaptations: hemodynamic responses were not different (p > 0.05), regardless of the assessment session; and chronicles: changes in lipidic profile favors G_RTBFR_, which significantly lower level of total cholesterol (p = 0.024), triglycerides (p = 0.002) and LDL (p = 0.030) compared to G_TRT_. RTBFR and TRT induced a similar hemodynamic adaptation in PWH, with no significant risks of increased cardiovascular stress. Additionally, RTBFR promoted better chronic adequacy of lipid profile than TRT. Therefore, RTBFR presents a safe resistance training alternative for PWH.

**Trial registration:** ClinicalTrials.gov ID: NCT02783417; Date of registration: 26/05/2016.

## Introduction

Antiretroviral therapy (ART) increased life expectancy in people diagnosed with Human Immunodeficiency Virus (HIV)^1,2^. However, cardiometabolic disorders such as dyslipidemia, insulin resistance, diabetes, hypertension, and increased inflammatory state are frequently observed amongst people living with HIV (PWH)^3^. The etiology of the abovementioned cardiometabolic abnormalities is complex and multifactorial and might be related to the inflammatory effects caused by the virus infection itself and/or by the ART treatment^4^. As a consequence, PWH exhibits an augmented cardiovascular risk^3,5,6^.

To counteract the adverse metabolic side effects of ART and infection, PWH is therefore encouraged to adopt a healthy lifestyle, with complementary physical exercise therapies^7^. Indeed, regular physical exercise, including traditional resistance training (TRT) in PWH, is recommended as an adjuvant therapy once it improves cardiovascular health^8^, lipid profile^9^, cardiorespiratory fitness, muscle strength, and body composition^10^. Additional benefits such as muscle hypertrophy are conferred by TRT at high intensities (i.e., 70% or more of one repetition maximum [1RM])^11^. However, intensities of this magnitude may be contraindicated for debilitated PWH, who often have physical incapacities and/or high cardiovascular risk^4,12,13^.

**Figure 1:**
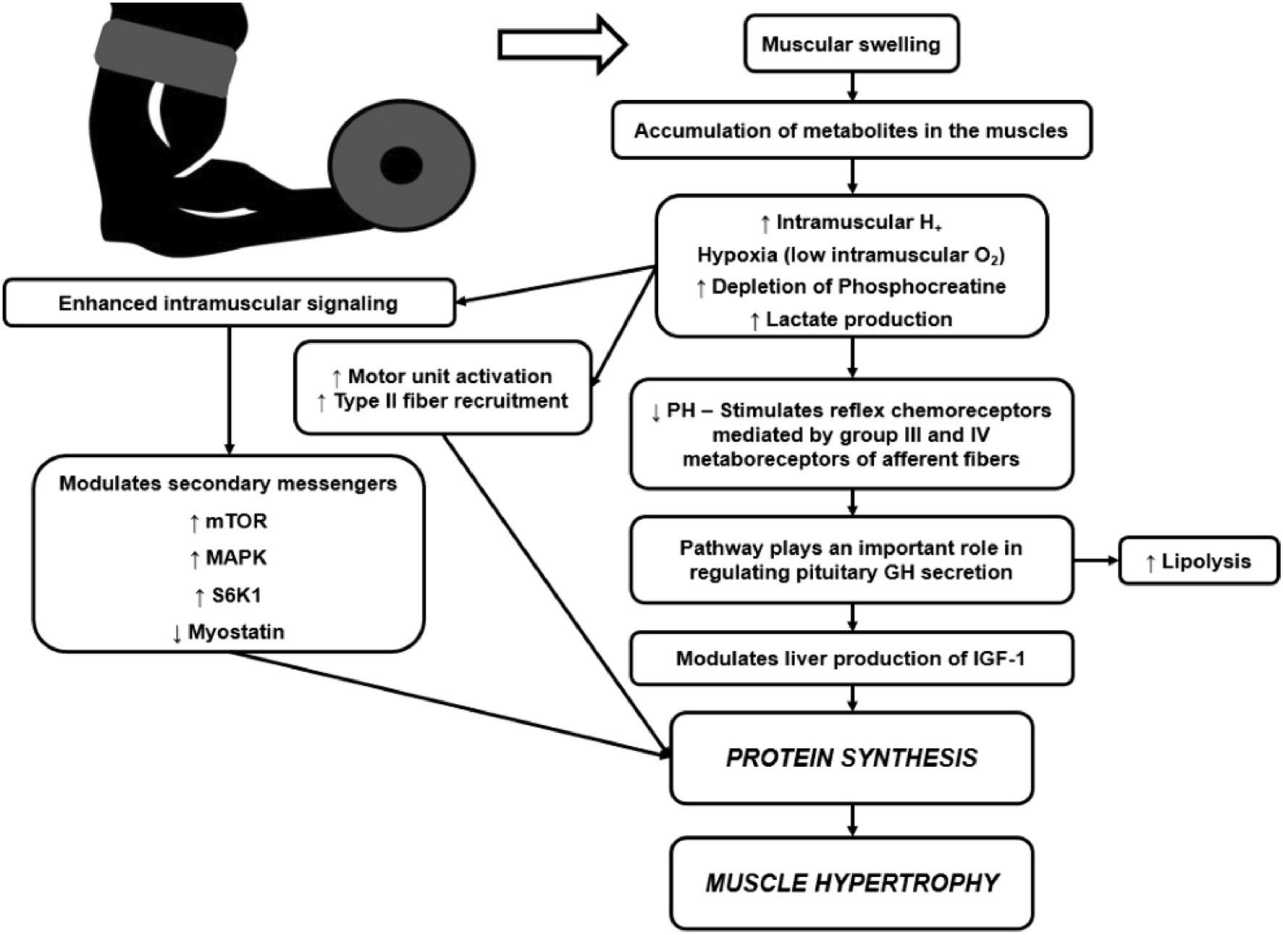
Mechanisms responsible for the efficiency of the RTBFR. H: hydrogen; O_2_: oxygen; GH: growth hormone; IGF-1: insulin-like growth factor I; mTOR: mammalian target of rapamycin; MAPK: mitogen-activated protein-kinase; S6K1: mitogen-stimulated protein kinase p70 ribosomal protein S6 kinase 1.

Resistance training with blood flow restriction (RTBFR) might configure an alternative approach to high-intensity TRT. This method is performed at low intensities (20% to 40% 1RM)^14,15^ because the ability to express strength is suppressed by restrictive cuffs on practitioners’ limbs. These cuffs evoke a considerable vascular stress^16^ which arises from both exogenous mechanical compression of the vessel and endogenous muscular compression. The resultant compression limits the capacity to exercise at high intensities and triggers several physiological mechanisms depicted in Fig. 1, as explained previously^17–19^.

The RTBFR increases muscle strength and muscle mass similar to the high-intensity TRT both in healthy^20^ and in PWH^4^ samples. Our previous founds demonstrate that are no significant differences in the effects of the two training methods (RTBFR and TRT) on muscle function and its morphology^4^. Although the methods strategically differ in terms of load, frequency, and ischemia, there is no difference in terms of training volume and muscle increase^4^. So, this study only looks at cardiovascular data in the same subject group (previously published) to examine the safety of these two types of training. Although RTBFR has been applied in diverse populations^21,22^; its safety regarding the acute hemodynamic responses is not yet fully established, and available results are conflicting. For example, some authors have demonstrated a superior increase in heart rate and blood pressure during RTBFR compared to TRT^23^. Others showed an equal or even a slighter response in the aforementioned variables following an RTBFR in comparison with TRT^12,24^. In terms of chronic effects of RTBFR on cardiovascular risk factors, studies results are attractive regarding blood pressure^25^ and metabolic profile^22,26,27^ for HIV-seronegative individuals. However, those researches looking for the effects of RTBFR on chronic cardiometabolic and hemodynamic factors in PWH are limited despite the high prevalence of systemic hypertension amongst this population^28^.

Thus, our study purposes were (i) to compare the safety of acute response of RTBFR and TRT on hemodynamic variables (heart rate [HR], blood pressure [BP], and double product [DP]) in PWH; and (ii) to verify the chronic hemodynamic and metabolic adaptations of RTBFR and TRT in PWH after 12 weeks of training. We hypothesize that both RTBFR and TRT lead to a similar acute response of hemodynamic variables. At least in healthy subjects, both types of training (RTBFR and TRT) there is a similar acute blood lactate elevation, and equal or lower hemodynamic responses in RTBFR compared to TRT^12^.

## Methods

### Participants

A non-probabilistic sample (n = 14, 57% women [n = 8]) of adults with PWH participated in the study. Inclusion criteria were: seropositive for HIV; aged between 30 and 60 years; not be pregnant; do not participate in any regular physical exercise program for at least three months; regular treatment with ART; body weight variation < 10% in the last six months; ankle/brachial index between 0.91 and 1.30; and have a physician clearance to integrate the exercise program. Participants were excluded if they presented symptoms that contraindicated their permanence in the training program (CD4+ T lymphocytes < 200 cells/mm^3^ and viral load > 100,000 copies of RNA/mL); cardiovascular disease symptoms of or opportunistic infections; and if they missed more than nine non-consecutive training sessions (> 25%).

### Experimental design

**Figure 2:**
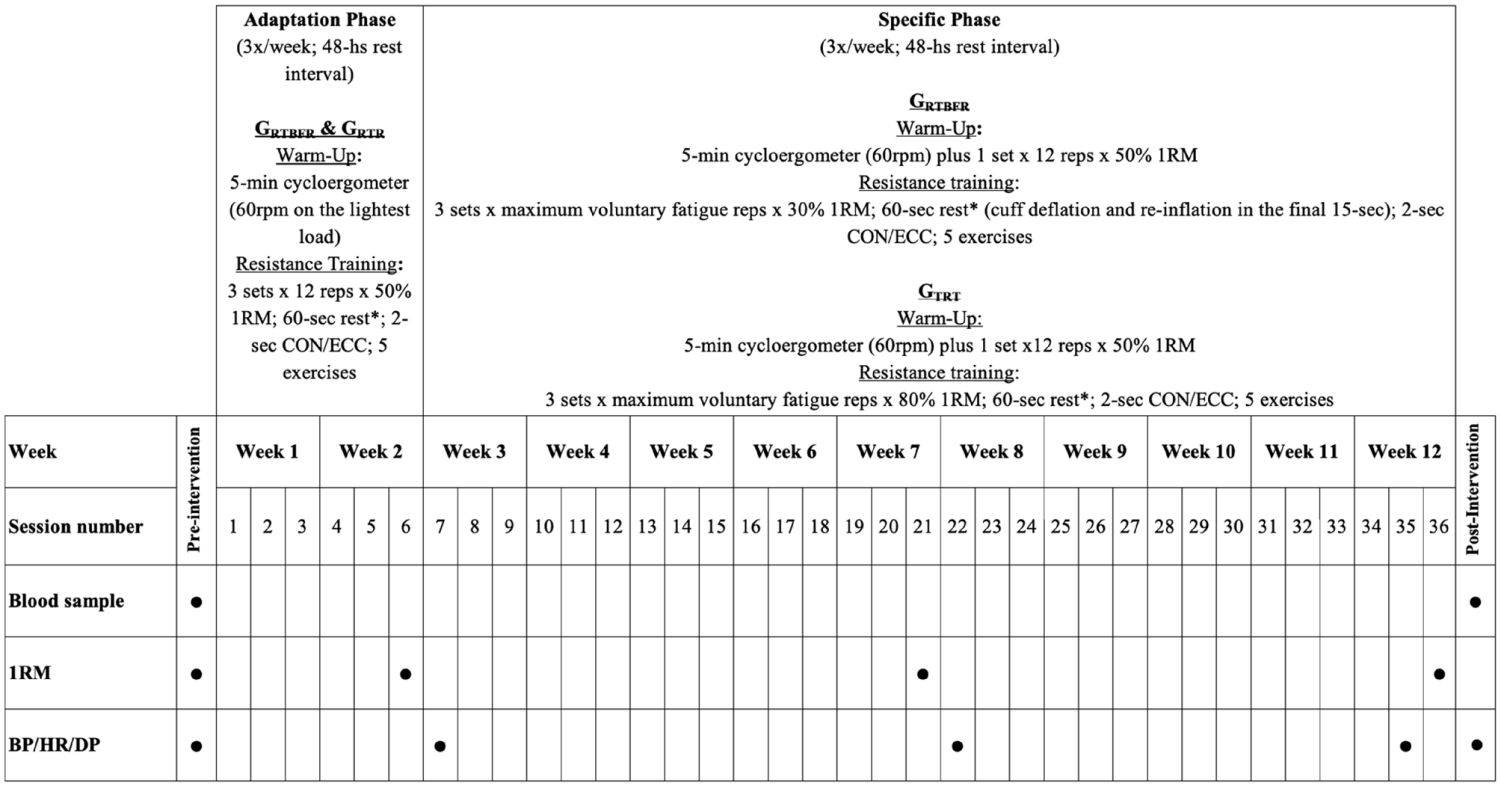
Training protocol and assessments during the intervention period. CON = concentric; ECC = eccentric; 1RM = one repetition maximum; BP = blood pressure; HR = heart rate; DP = double product; * = for each exercise and muscle grouping (upper or lower); A 3-min rest occurred in the transition between upper to lower limbs exercises.

This randomized controlled trial was carried out between August 2015 and February 2017 and included two intervention groups (TRT and RTBFR). Eligible participants were personally recruited during their clinic’s routine appointments in the Clinical Hospital Medical School at Ribeirao Preto/University of São Paulo; and in the Municipal Health Departments of the cities of Ribeirao Preto and Batatais, Southeast Brazil. There was a place for a single appointment in which participants provide sociodemographic information and they were assessed for cardiometabolic (ankle/brachial index and blood flow restriction [BFR]) and physiological parameters. After, participants were assessed for anthropometric and 1RM. To minimize errors, all measurements were performed by a single trained technician to limit measurement errors. The participants were equally and randomly distributed in quartiles according to the initial 1RM test (muscle strength levels) in a group of resistance training with blood flow restriction (G_RTBFR_; n = 7) or a group of traditional resistance training (G_TRT_; n = 7) (Fig. 2). The G_RTBFR_ exercise at 30% of 1RM, as recommended by Patterson to optimize muscle hypertrophy (intensity between 20 and 40% of 1RM)^14^; and the G_TRT_, at 80%, as recommended by the American College of Sports Medicine to optimize hypertrophy (intensity above 70% of 1RM^11^. In the G_RTBFR,_ cuffs were deflated between sets and exercise intervals to restore normal limb circulation, as additional care for PWH. This intermittent BFR favors a better rate of perceived exertion^29^.

### Measurements

#### Sociodemographic

Sociodemographic and clinical information (year of HIV diagnosis, ART treatment (duration and pharmacological composition), use of medication for hypertension, comorbidities, and risk factors for thrombosis were gathered via interviews and patients’ clinical files. Patients’ clinical files access was authorized by the Hospital Ethics Committee.

#### Brachial-ankle index

Systolic blood pressures (SBP) were obtained in 8 anatomical regions (posterior tibial artery and dorsal artery of the foot of both lower limbs; and brachial and radial arteries of both upper limbs) while participants were at supine position^30^. Measures were gathered with an aneroid sphygmomanometer (Premium^®^, Sao Paulo, Brazil) and a vascular doppler probe (DV 600, Martec Med^®^, Ribeirao Preto, Brazil). The ankle-brachial index (ABI) was obtained (for both sides) by the ratio of the systolic blood pressure (SBP) measured at the ankle to that measured at the brachial artery. For security considerations, ankle/brachial index lower than 0.91 or higher than 1.30 indicated the presence of peripheral obstructive arterial disease^30^. Then, the training was not done.

#### Blood flow restriction

The pressure to complete blood flow restriction (100% BFR, mmHg) was assessed using a Doppler vascular probe (DV-600; Marted, Ribeirão Preto, São Paulo, Brazil) and specific lower (170 × 900 mm) and upper body cuffs (70 × 730 mm) (Missouri^®^, PAIS). Detailed procedures are described elsewhere^4^. In brief, participants were lying down in the supine position, and cuffs were fixed on the participant’s limbs and inflated until the point where auscultatory monitored pulses were interrupted. Upper and lower body pressures at these points were used as 100% BFR and applied during training sessions for the G_RTBFR_. Higher BRF pressure (> 80%) has a greater muscle activation compared to milder intensities (40 or 60%)^31^.

For upper limbs, cuffs were attached to the proximal region, and the Doppler’s probe was placed over the radial artery. For lower limbs, cuffs were attached around the thighs at the inguinal fold region, and the Doppler’s probe was placed over the tibial artery. Upper and lower body BFR were measured subsequently.

#### One repetition maximum test

The 1RM was estimated following the Brzycki (1993) protocol. According to this, participants should perform proper repetitions in terms of velocity and range of motion until the failure, without exceeding 10 repetitions^32^. Based on the number of repetitions executed, the resistance (weight) is reduced or augmented to allow one to 10 repetitions. Participants had up to three attempts, with a 3-min rest interval between them. The test was carried out during the pre-intervention period and in the training sessions number 6, 21, and 36 (Fig. 2). At each point, exercise intensity was corrected.

#### Hemodynamics

Before each training session, heart rate and blood pressure were measured according to^33^ and Brazilian Society of Hypertension procedures^34^. All hemodynamic measures were always executed by the same investigator (TCA).

##### Heart rate

Resting heart rate (RHR) was measured after five-minute resting in the sitting position using the heart rate monitor (Polar FT7^®^; Embu das Artes/Brazil). It was also measured immediately post exercises.

##### Blood pressure

Resting SBP and diastolic blood pressure (DBP) were measured immediately after the RHR procedure (rest BP), using a stethoscope and aneroid sphygmomanometer (Premium^®^; Barueri, Brazil). Participants remained seated with the left arm supported at the heart level. Blood pressure was measured twice one minute apart. Additional measurements were taken if differences were higher than 5 mmHg for both SBP and DBP. The mean values between the two closer attempts were assumed as final SBP and DPB, as recommended^11^. The presence of hypertension was assumed if SBP ≥ 140 mmHg and/or DBP ≥ 90 mmHg were observed on two different days and/or if participants were undertaking antihypertension drugs^11^. BP was also measured between 20 and 30 s after the last repetition of the triceps exercise (intermediate BP), and between 20 and 30 s after the last repetition of the last set of the quadriceps exercise (post-exercise BP).

##### Double product

From SBP, DBP, and HR, the additional parameters Mean Blood Pressure (MBP; [SBP + (2 * DBP)]/3)^35^ and DP (HR * SBP)^36^ were computed. Hemodynamic parameters were assessed pre and post-intervention at rest, and post each exercise in sessions numbers 7, 22, and 35 (Fig. 2).

#### Metabolic profile

Lipidic profile (total cholesterol [TC], triglycerides [TG], low-density lipoprotein [LDL] and high-density lipoprotein [HDL]) and glycaemia (blood glucose) were determined pre-and post-training (Fig. 2). Blood samples were taken after 12 h of fasting and the enzymatic colorimetric method, with specific commercial kits (Wiener Lab^®^; Sao Paulo/Brazil), was used to get results. The lipidic profile was classified according to the NCEP ATP III and the dyslipidemia was confirmed when TC > 200 mg/dL and/or HDL < 40 mg/dL and/or TG > 150 mg/dL and/or LDL > 130 mg/dL^37^.

#### Resistance training protocols

Intensities for each group were established based on 1RM estimation. Each exercise session for both groups had four exercises executed one at a time in the following sequence: arm curl (biceps); arm extension (triceps); unilateral leg curl (hamstring); and leg extension (quadriceps). Resistance exercises were performed in an Athletic Way Training Station, with two independent 180 kg load columns. The training session was only initiated after getting vital signs within acceptable values (i.e., SBP ≤ 140 mmHg and DBP ≤ 90 mmHg)^11^.

##### Protocols

The 36 training sessions were divided into adaptation and specific phases. The adaptation phase lasted six sessions and was equal regardless of the group. In this phase, sessions had a 5-min warm-up period on a stationary cycle ergometer (load up to 60 rpm) plus resistance exercises (1 set × 12 repetitions × 50% 1RM). The 30-specific training sessions were specific according to the groups but the warm-up was similar (5-min, stationary cycle ergometer, load up to 60 rpm, plus one set of each exercise). Details about the training protocols are depicted in Fig. 2.

##### Training volumes

The volume of each exercise and each session (sum of the four exercises) were performed at the end of the adaptation phase (6th session), 7th week (21st session) and at the end of the 12th week (36th session). Thus, the total training load (nº of series × nº of reps × weight [kg]) was compared in three moments, at the 6th, 21st, and 36th sessions^4^.

### Energy intake

Because diet might impact training responses, participants were assessed for nutrition. The 24-h food record was used to determine the energy intake from food consumption, and it was applied on two weekdays and one weekend day, at the beginning and at the end of the study (3rd and 11th week). Participants registered all consumed products in detail. The first day of the 24-h food record was the day before the patient's assessment^38^. The chemical analysis of energy intake (DietPro^®^, version 5i) was performed with the nutritional coefficients of the Brazilian Food Composition Table (TACO)^39^ and with the National Nutrient Database for Standard Reference (USDA)^40^. The average of the 3 days of energy and macronutrient intake (% of the total calorie value) was considered.

### Statistical analysis

Power calculation was computed a priori using the software G*Power version 3.1. Considering a repeated measures design, for the within-between interaction, the sample size revealed that 24 subjects (12 per group) were required to detect a medium effect size (f = 0.25). The other parameters considered were a power of 80%, an error type I of 0.05, three measurements (7th, 22nd, and 35th session), two groups (RTBFR and TRT), and assuming a correlation equal to 0.60, and a no sphericity correction of 1.0. Data normality was checked with Shapiro–Wilk test. Descriptive statistics (measures of central tendency and dispersion) were used to describe the sample. At baseline, between groups comparisons (RTBFR *versus* TRT) were verified using an independent t-test, chi-square, and Mann–Whitney U test, as appropriate. Training volume comparisons according to exercise groups were checked using the independent t-test to ensure that any differences in responses between training types were not due to differences in training volume. Group changes over time (i.e., post–pre-intervention for metabolic variables) were ascertained using paired t-test or Wilcoxon test. Acute and chronic changes were adjusted for antihypertensive treatment in hemodynamic variables during each intragroup session and comparisons between groups were performed by GLM (group-by-time interactions). The SIDAK post hoc test was selected to verify between groups and moments differences when significant F values were found in the main effect comparisons. The delta of the alteration of the variables was calculated through the difference between the values after and pre-exercise and intervention, to verify the magnitude of the alteration provided by each training method on all the studied variables. To verify the impact of training methods on all studied variables the effect size (ES) was calculated ([Post-test mean – pre-test mean]/pretest standard deviation)^41^ and classified (trivial: < 0.50; small: 0.50–1.25; moderate: 1.25–1.90 and large: > 2.00 for untrained individuals) according to Rhea (2004). All analyses were performed assuming statistical significance previously (p < 0.05) on SPSS 20.0.

### Ethics approval

The study was approved by the Research Ethics Committee of the University of Sao Paulo School of Nursing at Ribeirao Preto (EERP/USP). It followed the recommendations that govern research involving human beings, which are also in accordance with the Declaration of Helsinki. This study was registered in ClinicalTrials.gov ID: NCT02783417, under protocol ID: 44195315.6.0000.5393. Date of registration: May 26, 2016.

### Consent to participate

Informed consent was obtained from all individual participants included in the study.

## Results

**Figure 3:**
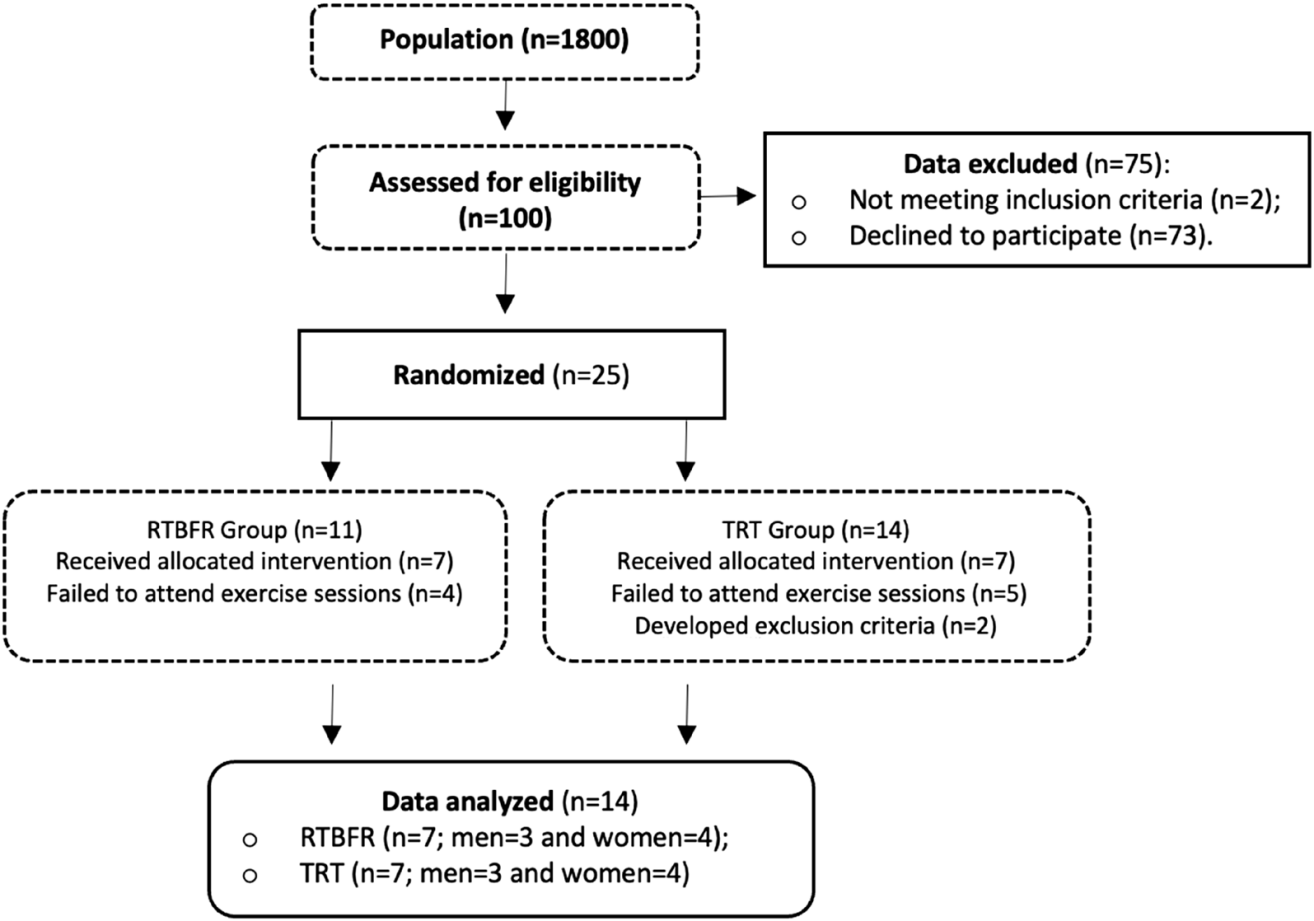
Study flowchart.

One hundred PWH were invited to integrate the study. Of those, 2 participants did not meet the inclusion criteria and 73 declined to participate. At the end of recruitment, 25 PWH were randomly allocated to one of the two exercise groups (G_RTBFR_:11; G_TRT_: 14). At the end of the protocol, each exercise group had 7 participants (Fig. 3).

Table 1 shows baseline characteristics and between-groups comparisons. At baseline, groups were not different for any considered variable. Nevertheless, the G_TRT_ tended to be different in TG compared to the G_RTBFR_ group (p = 0.050).

**Table 1:** Descriptive analysis and differences test between groups at baseline. *NOTE*: hemodynamic variables at rest.

	Total subjects (n = 14)				Difference test*	*p* value
	G_RTBFR_ (n = 7)		G_TRT_ (n = 7)			
	Mean ± SD	95% CI	Mean ± SD	95% CI		
Age (years)	45.4 ± 6.0	39.9–50.9	49.0 ± 7.9	41.7–56.3	− 0.955	0.359
Diagnosis of HIV (months)	156.8 ± 112.7	52.6–261.1	135.9 ± 93.9	49.0–222.7	0.379	0.711
**Anthropometry**						
Body mass index (kg/m^2^)	25.9 ± 4.1	22.1–29.6	28.0 ± 6.1	22.4–33.7	− 0.77	0.456
**Hemodynamic parameters**						
SBP (mmHg)	109.3 ± 10.2	99.9–118.7	117.1 ± 7.6	110.2–124.1		0.165
DBP (mmHg)	73.6 ± 8.5	65.7–81.5	81.4 ± 10.7	71.5–91.3	− 1.521^U^	0.154
MBP (mmHg)	85.5 ± 8.7	77.4–93.5	93.3 ± 8.6	85.4–101.3	− 1.699	0.115
HR (bpm)	78.3 ± 11.4	67.8–88.8	85.4 ± 16.6	70.1–100.7	− 0.941	0.365
DP (bpm·mmHg)	8625.0 ± 1981.0	6792.3–10,457.7	10,078.6 ± 2391.4	7866.9–12,290.2	− 1.238	0.239
**Metabolic profile**						
TC (mg/dL)	165.2 ± 19.8	144.4–186.0	188.0 ± 33.7	152.7–223.3	− 1.432	0.183
HDL (mg/dL)	50.9 ± 16.5	33.6–68.2	41.8 ± 13.1	28.1–55.6	1.053	0.317
LDL (mg/dL)	91.3 ± 12.0	78.7–103.9	114.2 ± 24.8	88.2–140.3	− 2.040	0.069
TG (mg/dL)	115.3 ± 63.1	49.1–181.6	182.0 ± 18.5	159.0–205.0	− 2.263	0.050
Glycaemia (mg/dL)	86.2 ± 8.6	77.2–95.2	89.8 ± 2.4	86.8–92.8	− 0.910	0.387
**Health conditions**						
Hypertension, (n; %)	3 (42.9)		3 (42.9)			0.704
Dyslipidemia, (n; %)	2 (28.6)		5 (71.4)			0.143
Type II Diabetes, (n; %)	0 (0.0)		1 (14.3)			0.500
**Treatment**						
Durantion of ART (months)	113.4 ± 90.1	30.1–196.7	97.4 ± 58.0	43.8 to 151.1	0.395	0.700
Antihypertension (n; %)	3 (42.9)		3 (42.9)			0.704

G_RTBFR_: group of resistance training with blood flow restriction; G_TRT_: group of traditional resistance training; HIV: human immunodeficiency virus; 95% CI: confidence interval; ART: antiretroviral therapy; (m): meters; (kg): kilograms; (kg/m^2^): kilograms/meters square; (cm): centimeters. SBP: systolic blood pressure; DBP: diastolic blood pressure; MBP: mean blood pressure; HR: heart rate; DP: double product; TC: total cholesterol; HDL: high-density lipoprotein cholesterol; LDL: low-density lipoprotein cholesterol; TG: triglycerides. *t-test for parametric variables. ^U^: Mann–Whitney for non-parametric variables.

The BFR for training in G_RTBFR_ were 135.7 ± 12.7 and 124.3 ± 15.1 mmHg, for the upper and lower body, respectively.

### Acute responses to exercise

#### Within groups comparisons: G_RTBFR_

**Figure 4:**
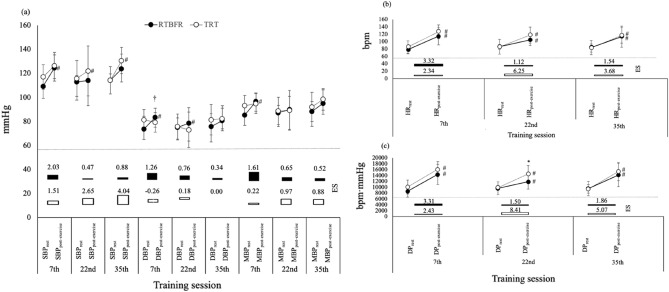
Within and between-group comparison for acute hemodynamic responses (rest *versus* post-exercise sessions) controlled for antihypertensive treatment. ^#^p < 0.05: Statistically significant difference between rest and immediately post-exercise (within groups comparisons; paired t-test or Wilcoxon test). *p < 0.05: Statistically significant difference between groups (general linear models-GLM with SIDAK post hoc test adjusted for antihypertensive treatment). ^†^p < 0.05: Statistically significant group-by-time interactions. (**a**) Blood pressure; (**b**) heart rate; (**c**): double product. RTBFR: resistance training with blood flow restriction; TRT: traditional resistance training; ES: effect size; SBP: systolic blood pressure; DBP: diastolic blood pressure; MBP: mean blood pressure; HR: heart rate; DP: double product. Obs: acute intra-exercise hemodynamic responses in the middle of the session (intermediate) were not displayed for figure resolution purposes. These values were displayed in Table S1.

Acute effects of exercise on training groups were performed for hemodynamics in sessions 7th, 22nd, and 35th (Fig. 3). In the 7th training session, the hemodynamic variables SBP (∆ = 20.7 mmHg; p = 0.021), DBP (∆ = 10.7 mmHg; p = 0.042), MBP (∆ = 10.0 mmHg; p = 0.019), HR (∆ = 37.9 bpm; p = 0.002), and DP (∆ = 6555.0 bpm.mmHg; p = 0.001) changed significantly from pre- to post-exercise session in the G_RTBFR_ (Fig. 4 and Table S1). In the 22nd session, the G_RTBFR_ showed a statistically significant increase in the DBP (∆ = 7.9 mmHg; p = 0.033), HR (∆ = 22.3 bpm; p = 0.034) and DP (∆ = 3314.8 bpm.mmHg; p = 0.019) and in the 35th session, the G_RTBFR_ showed a statistically significant increase for HR (∆ = 29.8 bpm; p = 0.007) and DP (∆ = 4538.1 bpm.mmHg; p = 0.002) (Fig. 4; Table S1).

#### Within groups comparisons: G_TRT_

In the 7th training session the G_TRT_, significantly increments were observed for HR (∆ = 38.9 bpm; p = 0.001) and DP (∆ = 5986.3 bpm.mmHg; p = 0.001). In the 22nd session the G_TRT_ for SBP (∆ = 14.3 mmHg; p = 0.042), HR (∆ = 38.1 bpm; p = 0.001) and DP (∆ = 3644.8 bpm.mmHg; p = 0.001) and in the 35th session showed a statistically increments for SBP (∆ = 21.4 mmHg; p = 0.004), HR (∆ = 40.5 bpm; p = 0.001) and DP (∆ = 7235.2 bpm.mmHg; p = 0.001) (Fig. 4; Table S1).

#### Effect size

The G_RTBFR_ presented a large ES classification for all hemodynamic parameters [SBP (ES = 2.03), DBP (ES = 1.26), MBP (ES = 1.61), HR (ES = 3.32), and DP (ES = 3.31)] in the 7th training session (Fig. 3). Similarly, the ES classification for hemodynamics in the G_TRT_ was moderate to large [SBP (ES = 1.51), HR (ES = 2.34), and DP (ES = 2.43)] during the same session (Fig. 4; Table S1).

In the 22nd session, the G_TRT_ presented large ES for SBP (2.65), HR (6.25), and DP (8.41) while the G_RTBFR_ presented a moderate ES classification only for DP (1.5). In the 35th session, the G_TRT_ sustained a large ES for SBP (4.04), HR (3.68), and DP (5.07) whereas the G_RTBFR,_ had a moderate ES for HR (1.54) and DP (1.86) (Fig. 4; Table S1).

#### Between groups comparisons: G_RTBFR_ versus G_TRT_

Between groups comparisons for acute hemodynamics did not show any significant difference at the different moments of assessment (7th, 22nd, and 35th sessions) (Fig. 4).

### Chronic responses to exercise

#### Within-group comparisons for G_RTBFR_ and G_TRT_

**Figure 5:**
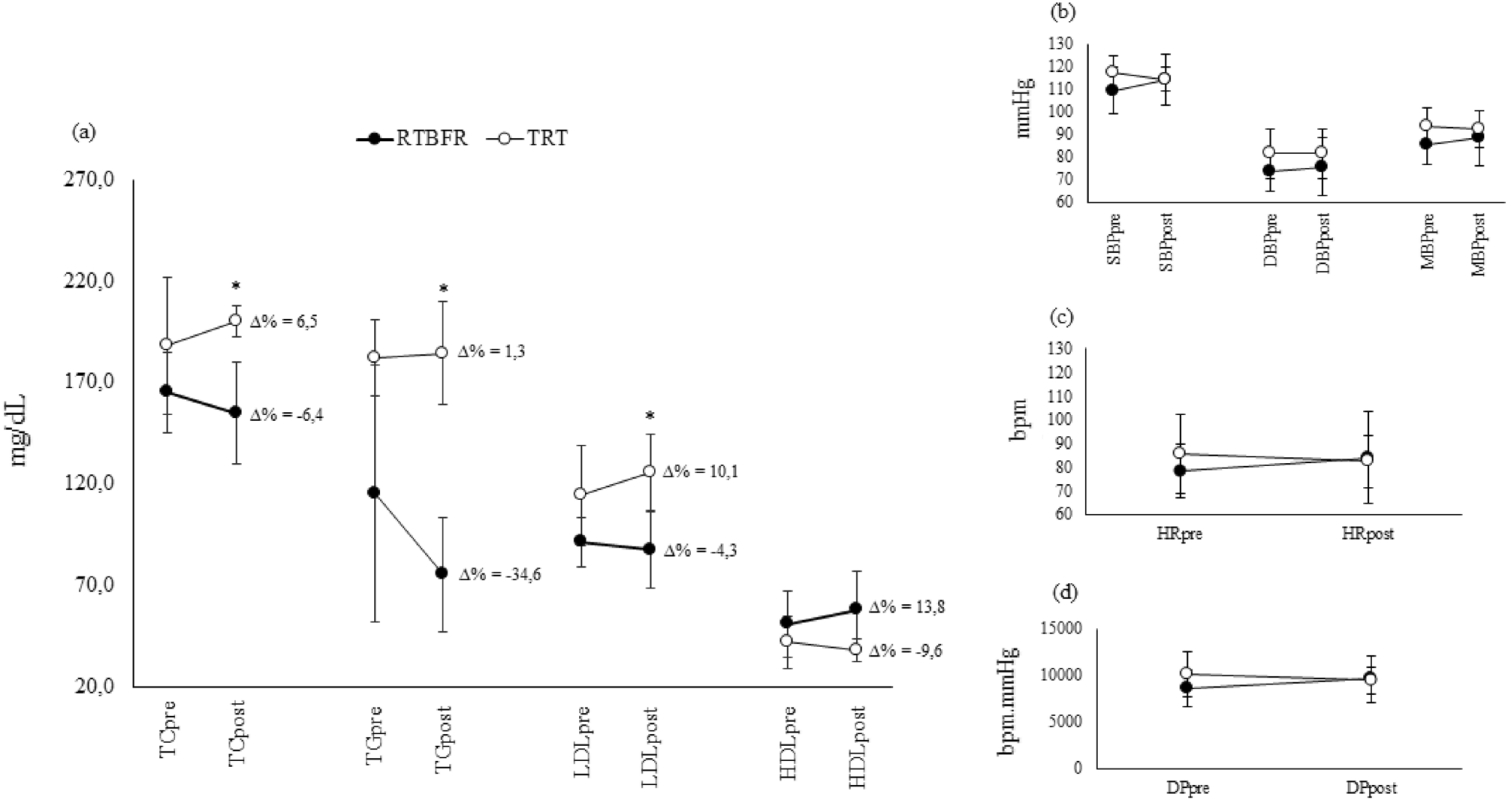
Chronic effects between and within groups (pre vs post-training) for lipids (**a**), blood pressure (**b**), heart rate (**c**), and double product (**d**). *p < 0.05: Statistically significant difference between groups (general linear models-GLM with SIDAK post hoc test) cardiovascular variables at rest. RTBFR: resistance training with blood flow restriction; TRT: traditional resistance training; SBP: systolic blood pressure; DBP: diastolic blood pressure; MBP: mean blood pressure; HR: heart rate; DP: double product; TC: total cholesterol; HDL: high-density lipoprotein cholesterol; LDL: low-density lipoprotein cholesterol; TG: triglycerides.

Regardless to exercise groups and outcomes, there was no significant change over time for G_RTBFR_ on TC (165.2 ± 19.8 to 154.6 ± 25.0 mg/dL; ∆ = − 6.4%; p = 0.232), TG (115.3 ± 63.1 to 75.4 ± 28.2 mg/dL; ∆ = − 34.6%; p = 0.230), LDL (91.3 ± 12.0 to 87.4 ± 18.5 mg/dL; ∆ = − 4.3%; p = 0.745), and HDL (50.9 ± 16.5 to 57.9 ± 18.9 mg/dL; ∆ = 13.8%; p = 0.148), and for G_TRT_ on TC (188.0 ± 33.7 to 200.3 ± 7.6 mg/dL; ∆ = 6.5%; p = 0.825), TG (182.0 ± 18.5 to 184.3 ± 25.4 mg/dL; ∆ = 1.3%; p = 0.928), LDL (114.2 ± 24.8 to 125.7 ± 18.5 mg/dL; ∆ = 10.1%; p = 0.968), and (41.8 ± 13.1 to 37.8 ± 5.9 mg/dL; ∆ = − 9.6%; p = 0.844) (Fig. 5; Table S1).

#### Between-group comparisons: G_RTBFR_ versus G_TRT_

Between groups comparisons for chronic exercise effects (pre- and post-training) for lipidic and hemodynamic indexes according to exercise interventions are depicted in Fig. 5. Results showed that after intervention, G_RTBFR_ compared to G_TRT_ had a lower mean for TC (154.6 ± 25.0 *versus* 200.3 ± 7.6 mg/dL, respectively; p = 0.024), TG (75.4 ± 28.2 *versus* 184.3 ± 25.4 mg/dL, respectively; p = 0.002) and LDL-cholesterol (87.4 ± 18.5 *versus* 125.7 ± 18.5 mg/dL, respectively; p = 0.030) (Fig. 5). Although group-by-time interactions were not significant for lipid (TC: p = 0.422; TG: p = 0.352; LDL-cholesterol: p = 0.531; and HDL-cholesterol: p = 0.446) and hemodynamic indexes (SBP: p = 0.114, DPB: p = 0.607: MBP: p = 0.342; HR: p = 0.496; DP: p = 0.314).

Groups exhibit similar training loads at sessions number 6st (G_RTBFR_: 1042.9 *versus* G_TRT_: 1050.0; p = 0.972) 21st (G_RTBFR_: 3216.8 *versus* G_TRT_: 4292.3; p = 0.259) and 36th (3703.5 *versus* 4446.9; p = 0.319), indicating that training volume across groups were not different. Table S2 shows the weight, no of repetitions, and the load for both groups in adaptation and specific phases. Similarly, energy and macronutrient intake were similar between groups at different assessment moments (Table S1).

## Discussion

This randomized controlled trial aimed to compare the safety of acute (rest versus post-exercise conditions) and chronic (before and after 12 weeks of training) responses to hemodynamic (HR, BP, and DP) and lipid variables from two strength training approaches (RTBFR versus TRT) in the PWH. The results in muscle strength and volumes of these patients have already been demonstrated in our previous study^3^. The study’s main findings showed that both RTBFR and TRT are similar in terms of acute impact on hemodynamics, suggesting that RTBFR does not prompt an unwarranted hemodynamic response. On the chronic effect, RTBFR showed better adaptation to training in all hemodynamic parameters, after a relatively shorter period (7th week), suggesting a faster and notably significant response to training to the PWH more weakened. Moreover, RTBFR provoked a superior lipidic profile adaptation in PWH compared to TRT. According to the best of our knowledge, the inexistence of other studies including similar training approaches by PWH^4^ difficult between studies comparisons.

The acute hemodynamic response during a resistance training bout is controlled by the brain stem which important neural mechanism of central command, taking into account a combination of the mechanical changes in muscles and tendons (i.e., mechanoreflex) and the accumulation of metabolites in the active contracting muscle (i.e. muscle metaboreflex)^42^. Both exercise protocols prompted a similar increase in HR and DP. The RTBFR induces an additional exogenous mechanical compression of blood vessels and an endogenous muscle compression, that together exacerbate blood pressure and HR responses during an RTBFR training session compared to TRT^16^. The physiological mechanism underlying this phenomenon is that the venous vascular occlusion augments metabolite accumulation, which stimulates muscle chemoreceptors to produce and release catecholamines through the stimulus of the sympathetic nervous system^43^. In RTBFR, there is a reduction in blood flow to the exercised muscle. Reduced blood flow during muscle contraction increases metabolic stress and may increase muscle or reflex pressure to the cardiovascular control center, causing exaggerated sympathetic nerve activity. Increased sympathetic activity can lead to increased peripheral vascular resistance and, consequently, increase DBP during exercise^44^. Moreover, other factors (e.g., active muscle volume, protocol characteristics including exercise intensity, number of exercises, sets and repetitions, cuff width, BFR compression pressure, releasing or maintaining cuffs pressure during intervals between sets) may contribute to an additional HR and BP intensification during RTBFR^14,43,45^. Surprisingly, in our findings, the RTBFR until the concentric failure (meaning a huge intensity/volume) did not evoke greater hemodynamic stress. It is possible to suggest that the cuff releasing during intervals between sets and exercises probably reduced the impact that exercise could exert on central nervous system responses, and consequently, on HR and SBP^43^.

Previous acute studies have shown that BP and HR responses induced by both RTBFR and TRT are similar for limbs involved in the training program, even when exercise protocols have involved just lower^12^ or both, upper and lower limbs^46^. Our results with PWH are aligned with those findings. Despite the lack of statistical differences, in the G_RTBFR_ group, the ES of hemodynamic variables exhibited a trend to decrease along with the study protocol. Conversely, an opposite trend was observed for the G_TRT_. We might suggest that this indicates an adaptation to the type of training triggered by attenuation of discomfort or pain felt by the G_RTBFR_ participants arising from metabolic acidosis^12,47^, which generally impacts increased hemodynamic responses.

In terms of chronic adaptations for hemodynamic responses, results between and within groups were similar. For the lipidic profile, there was observed a minimal positive variation favoring the G_RTBFR_ at the end of the protocol (i.e., LDL, TG, and TC reduction and HDL augmentation). These findings suggest that RTBFR might be useful to manage dyslipidemia induced by ART drugs. However, it is important to highlight that this result must be interpreted with caution due to the small sample size.

Resistance training improves cardiometabolic profile via lipoprotein lipase activity enzyme stimulation and thus, it may protect against the onset of health conditions such as atherosclerosis, metabolic syndrome, and cardiovascular diseases^13,48^. The lipoprotein lipase activity enzyme favors the exit of TG fatty acids from the adipose and muscle tissues, which increases TG catabolism and facilitates the removal of TG from the bloodstream^26^. As above mentioned, the G_RTBFR_ showed an improvement in the lipidic profile, which might be partially explained by an expressive blood secretion of growth hormone following the RTBFR^49,50^. It is speculated that the growth hormone signals protein synthesis by modulating insulin-like growth factor I (IGF-1)^22^. In addition, the growth hormone also increases lipolysis enhancing the reduction of visceral fat and positively affecting circulating lipids^22^, typically up-regulated in PWH. Figure 1 resumes the physiological pathways linked to the positive effects triggered by RTBFR.

An unexpected result was the lipidic profile deterioration at the end of the G_TRT_ protocol. The available literature highlights the improvement both in lipidic and metabolic profiles in PWH undergoing TRT^9,51^. However, it is common degradation of the lipid profile in PWH and therefore, any maintenance of lipids would already represent a clinical advantage. Despite it, the baseline TG values presented by the G_TRT_ (182 mg dL^−1^) were above the 150 mg dL^−1^ and it did not change at the end of the protocol. It is still unclear why there was a positive trend change in the TG level only in the G_RTBFR_. The increase in muscle mass may increase the elimination of TG in the circulation^51^. However, PWH with lipodystrophy are characterized by mitochondrial dysfunction and this may represent one of the underlying mechanisms for dyslipidemia in this population. Therefore, the TRT effect on mitochondrial biogenesis and lipid oxidation may not have been achieved in our study^9^.

Our study further monitored the chronic hemodynamic and metabolic effects of RTBFR and TRT considering similar training volumes. During the 12 weeks of intervention, the training intensities of both groups remained stable although the exercise loads were adjusted according to the 1-RM reassessments. Regardless of the lack of significant changes between groups, the G_RTBFR_ had a slightly lower HR and DP at the end of exercise sessions, suggesting an eventual tendency of superior hemodynamic stability, potentially masked by the small sample size.

Although the frequency of antihypertensive drugs was the same in both groups, we decided to adjust our analysis to it, due they exert a physiological impact on acute and chronic hemodynamic adaptations. This is especially true considering that many antihypertensive classes could be administered for different periods to participants^52^. Chronic administration of antihypertensive drugs would likely result in suitable BP before the start of the study. We detected 3 cases in each intervention group (RTBFR and TRT), but we did not have access to the information on the antihypertensive treatment duration. To solve this impasse, we proceed with data analysis without controlling for antihypertensive drugs, and the results did not change (data not shown). This is particularly important because cardiometabolic abnormalities, including hypertension, are highly prevalent amongst PWH^6^. Between studies, the comparison is difficult because there was only one study on RTBFR with similar aims to ours, but in this case, the sample was older adults with coronary artery disease. In this study, eight weeks of RTBFR (unilateral knee extension) induced a decrease in resting SBP, but, immediately after the training session, SBP increased of the same magnitude throughout the intervention period^25^.

To the best of our knowledge, this is the first study measuring the impact of RTBFR on hemodynamic and metabolic profiles in PWH. As far as we know, most studies using RTBFR include exercises for the upper or lower body hampering comparability against TRT (which normally involves upper and lower body exercises in the same session). The protocol adopted by us included two exercises for the lower and two for the upper body in the same session, which is more close to daily resistance training routines^53^, and requires a bigger muscle volume, impacting more exuberant acute hemodynamic adaptations^23,43^. Our results were pioneering in showing that whole body RTBFR did not induce a bigger hemodynamic response compared to TRT, demonstrating that the method seems safe from the circulatory system point of view in a particular clinically debilitated populations^54^. The fact that the training volume was controlled and that it was similar in both groups along the study protocol overcome the usual limitation (different training volumes across studies groups) pointed out in many studies looking at the acute and chronic effects of exercise interventions. Additionally, the random distribution of participants balanced according to 1RM ensured homogeneity between groups at baseline, strengthening the comparisons between training methods. Finally, the consideration of food intake along the protocol prevented an eventual intragroup dietary difference in the type of training/adaptation. In combination, all these methodological details ensure that there was no influence of baseline differences in terms of strength, training load (volume), and diet on the results.

Despite the small sample size, this is a very particular (and difficult) population to recruit and to carry on a randomized controlled trial as we did. Immunological disbalance, low income, and social exclusion are some of the barriers to PWH for the availability to participate and attend training sessions, alongside a higher prevalence of cognitive difficulties or depression^55^. Another limitation was the intermittent monitoring of BP instead continuous (i.e., rest and post exercises of the training session). This might have compromised the recording of pressure peaks during exercise performance, which could be above the ones observed immediately after the exercise series^12^.

The American College of Sports Medicine encourages PWH to perform TRT (2–3 times/week, 1–3 sets, 8–10 reps up to 60% of 1RM)^53^. However, the same institution assumes that evidence (and its level) that has supported these recommendations are very low^11^ reinforcing the need to verify the best resistance training approach exerting the superior health benefit. Depending on the HIV status (asymptomatic and symptomatic), care must be taken with the assessment of functional, hemodynamic, and metabolic parameters of PWH^7^. In asymptomatic patients, physiological parameters (blood pressure, heart rate, and oxygen consumption) are normal but there may be low aerobic impairment (if sedentary)^7^. When in the symptomatic phase, PWH has reduced aerobic capacity and muscle strength, the possibility of an increased resting heart rate and submaximal work rates, difficulties in adhering to exercise programs, and side effects related to antiretroviral therapy. In the stronger symptomatic phase status, there is a drastic reduction in aerobic capacity and muscle strength, in addition to a possible abnormal endocrine response at moderate and high-intensity work rates^7^. Both RTBFR and TRT induce muscle hypertrophy and promote desirable body composition changes in PWH^4^. The larger impact of RTBFR on lipid profile suggests the superiority of this method for PWH, whose population usually has dyslipidemia^3^, closely linked to an augmented cardiovascular risk. Although our study results failed in showing a chronic hypotensive effect of RTBFR, it was observed a trend toward lower hemodynamic stress in PWH compared to TRT. Considering the abovementioned, the RTBFR seems to be a safe strength training methodology for more fragile PWH.

The RTBFR showed no different performance than TRT, proving to be a safer alternative therapy by resistance training method for PWH. Frail patients could be favored by training with BFR instead TRT because it is performed at lower intensities, induced similar chronic and acute hemodynamic responses, and seems to be superior in terms of lipids regulation. Indeed, the impact of RTBFR on the serum-lipid profile suggests its cardioprotective effect. The clinical significance for the health of PWH is that integrative practices, such as resistance training, contribute to lesser susceptibility to chronic non-communicable diseases. Thus, we conclude that RTBFR can be considered a safe complementary therapy in the treatment of PWH.

## Supplementary Information

Supplementary Tables.

## Data Availability

The datasets generated during and/or analyzed during the current study are available from the corresponding author upon reasonable request.

## Abbreviations

1RM: One repetition maximum
BFR: Blood flow restriction
BP: Blood pressure
ART: Antiretroviral therapy
DBP: Diastolic blood pressure
DP: Double product
ES: Effect size
G_RTBFR_: Group of resistance training with blood flow restriction
G_TRT_: Group of traditional resistance training
HDL: High-density lipoproteins
HIV: Human immunodeficiency virus
HR: Heart hate
LDL: Low-density lipoproteins
MBP: Mean blood pressure
PWH: People living with HIV
RHR: Resting heart rate
RTBFR: Resistance training with blood flow restriction
SBP: Systolic blood pressure
TC: Total cholesterol
TG: Triglycerides
TRT: Traditional resistance training

## References

[1] dos Santos WR (2013). Impact of progressive resistance training in Brazilian HIV patients with lipodystrophy. J. AIDS Clin. Res..

[2] Smiley CL (2021). Estimated life expectancy gains with antiretroviral therapy among adults with HIV in Latin America and the Caribbean: A multisite retrospective cohort study. Lancet HIV.

[3] Stanley TL, Grinspoon SK (2012). Body composition and metabolic changes in HIV-infected patients. J. Infect. Dis..

[4] Alves TC (2021). Resistance training with blood flow restriction: Impact on the muscle strength and body composition in people living with HIV/AIDS. Eur. J. Sport Sci..

[5] Feinstein MJ (2019). On behalf of the American Heart Association Prevention Science Committee of the Council on Epidemiology and Prevention and Council on Cardiovascular and Stroke Nursing; Council on Clinical Cardiology; and Stroke Council Characteristics, Prevention, and Management of Cardiovascular Disease in People Living With HIV A Scientific Statement From the American Heart Association. Circulation.

[6] Non LR, Escota GV, Powderly WG (2017). HIV and its relationship to insulin resistance and lipid abnormalities. Transl. Res..

[7] Jaggers JR, Hand GA (2016). Health benefits of exercise for people living with HIV: A review of the literature. Am. J. Lifestyle Med..

[8] Ozemek C, Erlandson KM, Jankowski CM (2020). Physical activity and exercise to improve cardiovascular health for adults living with HIV. Prog. Cardiovasc. Dis..

[9] Lindegaard B (2008). The effect of strength and endurance training on insulin sensitivity and fat distribution in human immunodeficiency virus-infected patients with lipodystrophy. J. Clin. Endocrinol. Metab..

[10] O’Brien KK, Tynan A-M, Nixon SA, Glazier RH (2017). Effectiveness of Progressive Resistive Exercise (PRE) in the context of HIV: Systematic review and meta-analysis using the Cochrane Collaboration protocol. BMC Infect. Dis..

[11] Riebe D, Ehrman JK, Liguori G, Magal M, Medicine, A. C. of S (2018). ACSM’s Guidelines for Exercise Testing and Prescription.

[12] Poton R, Polito MD (2016). Hemodynamic response to resistance exercise with and without blood flow restriction in healthy subjects. Clin. Physiol. Funct. Imaging.

[13] Downs ME (2014). Acute vascular and cardiovascular responses to blood flow–restricted exercise. Med. Sci. Sports Exerc..

[14] Patterson SD (2019). Blood flow restriction exercise position stand: Considerations of methodology, application, and safety. Front. Physiol..

[15] Conceição MS, Ugrinowitsch C, Soares Conceição M (2019). Exercise with blood flow restriction: An effective alternative for the non-pharmaceutical treatment for muscle wasting. J. Cachexia Sarcopenia Muscle.

[16] Rossow LM (2012). Cardiovascular and perceptual responses to blood-flow-restricted resistance exercise with differing restrictive cuffs. Clin. Physiol. Funct. Imaging.

[17] Loenneke JP, Wilson GJ, Wilson JM (2010). A mechanistic approach to blood flow occlusion. Int. J. Sports Med..

[18] Loenneke JP, Fahs CA, Rossow LM, Abe T, Bemben MG (2012). The anabolic benefits of venous blood flow restriction training may be induced by muscle cell swelling. Med. Hypotheses.

[19] Scott BR, Slattery KM, Sculley DV, Dascombe BJ (2014). Hypoxia and resistance exercise: A comparison of localized and systemic methods. Sports Med..

[20] Farup J (2015). Blood flow restricted and traditional resistance training performed to fatigue produce equal muscle hypertrophy. Scand. J. Med. Sci. Sports.

[21] Nakajima T (2006). Use and safety of KAATSU training: Results of a national survey. Int. J. KAATSU Train. Res..

[22] Satoh I (2011). Kaatsu training: Application to metabolic syndrome. Int. J. KAATSU Train. Res..

[23] Scott BR, Peiffer JJ, Thomas HJ, Marston KJ, Hill KD (2018). Hemodynamic responses to low-load blood flow restriction and unrestricted high-load resistance exercise in older women. Front. Physiol..

[24] Moriggi R (2015). Similar hypotensive responses to resistance exercise with and without blood flow restriction. Biol. Sport.

[25] Kambič T, Novaković M, Tomažin K, Strojnik V, Jug B (2019). Blood flow restriction resistance exercise improves muscle strength and hemodynamics, but not vascular function in coronary artery disease patients: A pilot randomized controlled trial. Front. Physiol..

[26] FarzanehHesari A, Ebrahimi A, Azizi Zanjani M, Mahdavi S (2018). Effects of resistance training with and without blood flow restriction on cardiovascular risk factors in overweight females. Med. Lab. J..

[27] Souza DC (2020). The acute effect of a single resistance training session on the glycemic response among women with HIV/AIDS. Int. J. Exerc. Sci..

[28] Kaku S-A (2020). HIV and cardiovascular disease. Lancet HIV.

[29] Sinclair P, Kadhum M, Paton B (2022). Tolerance to intermittent vs continuous blood flow restriction training: A meta-analysis. Int. J. Sports Med..

[30] Norgren L (2007). Inter-society consensus for the management of peripheral arterial disease (TASC II). J. Vasc. Surg..

[31] Fatela P, Reis JF, Mendonca GV, Avela J, Mil-Homens P (2016). Acute effects of exercise under different levels of blood-flow restriction on muscle activation and fatigue. Eur. J. Appl. Physiol..

[32] Whisenant MJ, Panton LB, East WB, Broeder CE (2003). Validation of submaximal prediction equations for the 1 repetition maximum bench press test on a group of collegiate football players. J. Strength Cond. Res..

[33] Libardi CA (2015). Effect of concurrent training with blood flow restriction in the elderly. Int. J. Sports Med..

[34] Nobre, F. VI Diretrizes Brasileiras de Hipertensão. *Revista Brasileira de Hipertensão* Vol. 17, (2010).

[35] Moran D (1995). Calculation of mean arterial pressure during exercise as a function of heart rate. Appl. Hum. Sci..

[36] Kitamura K, Jorgensen CR, Gobel FL, Taylor HL, Wang Y (1972). Hemodynamic correlates of myocardial oxygen consumption during upright exercise. J. Appl. Physiol..

[37] Grundy SM (2004). Implications of recent clinical trials for the National Cholesterol Education Program Adult Treatment Panel III guidelines. Circulation.

[38] Monteiro JP (2010). Consumo alimentar: visualizando porções.

[39] NEPA, N. D. E. E. E. P. E. M. A. Tabela Brasileira de Composição de Alimentos–TACO. In *4*^*a*^* edição revisada e ampliada* (NEPA-UNICAMP, 2011).

[40] Food, U. S. & Administration, D. National nutrient database for standard reference Release 24. (2012).

[41] Madarame H, Nakada S, Ohta T, Ishii N (2018). Postexercise blood flow restriction does not enhance muscle hypertrophy induced by multiple-set high-load resistance exercise. Clin. Physiol. Funct. Imaging.

[42] Crisafulli, A., Marongiu, E. & Ogoh, S. *Cardiovascular Reflexes Activity and Their Interaction during Exercise* (2015)10.1155/2015/394183. 10.1155/2015/394183 PMC4628760 26557662

[43] Araújo JP (2014). The acute effect of resistance exercise with blood flow restriction with hemodynamic variables on hypertensive subjects. J. Hum. Kinet..

[44] Domingos E, Polito MD (2018). Blood pressure response between resistance exercise with and without blood flow restriction: A systematic review and meta-analysis. Life Sci..

[45] Centner C, Wiegel P, Gollhofer A, König D (2019). Effects of blood flow restriction training on muscular strength and hypertrophy in older individuals: A systematic review and meta-analysis. Sports Med..

[46] Neto GR (2015). Hypotensive effects of resistance exercises with blood flow restriction. J. Strength Cond. Res..

[47] Martín-Hernández J (2017). Adaptation of perceptual responses to low-load blood flow restriction training. J. Strength Cond. Res..

[48] Romancini JLH (2012). Níveis de atividade física e alterações metabólicas em pessoas vivendo com HIV/AIDS. Revista Brasileira de Medicina do Esporte.

[49] Takano H (2005). Hemodynamic and hormonal responses to a short-term low-intensity resistance exercise with the reduction of muscle blood flow. Eur. J. Appl. Physiol..

[50] Sato Y, Yoshitomi A, Abe T (2005). Acute growth hormone response to low-intensity KAATSU resistance exercise: Comparison between arm and leg. Int. J. KAATSU Train. Res..

[51] Yarasheski, K. E. *et al. Resistance Exercise Training Reduces Hypertriglyceridemia in HIV-Infected Men Treated with Antiviral Therapy* (2001). 10.1152/jappl.2001.90.1.133 11133903

[52] Feldman RD (2015). Intraclass differences among antihypertensive drugs. Ann. Rev. Pharmacol. Toxicol..

[53] Bushman B, Medicine, A. C. of S. (2017). ACSM’s Complete Guide to Fitness & Health, 2E.

[54] Hughes L, Paton B, Rosenblatt B, Gissane C, Patterson SD (2017). Blood flow restriction training in clinical musculoskeletal rehabilitation: A systematic review and meta-analysis. Br. J. Sports Med..

[55] Dos Santos AP (2019). Anthropometric cutoff points to identify lipodystrophy characteristics in people living with HIV/AIDS: An observational study. Nutr. Hosp..

