# Supplementary material for: Acute and chronic cardiometabolic responses induced by resistance training with blood flow restriction in HIV patients
Source: Sci Rep. 2022 Oct 10;12:16989. doi: 10.1038/s41598-022-19857-3 (PMC9550823; doi:10.1038/s41598-022-19857-3)
Supplement: Supplementary file 1 — Supplementary Tables. [file 41598_2022_19857_MOESM1_ESM.docx]

**SUPPLEMENTARY INFORMATION**

**Table S1** Analysis of differences between pre- and post-exercise session of hemodynamic, and pre- and post-training for metabolic variables and food intake variables

|  | **Participants (n=14)** | | | | | | | | | | |
| --- | --- | --- | --- | --- | --- | --- | --- | --- | --- | --- | --- |
|  | G_RTBFR_ (n = 7) | | | | |  | G_TRT_ (n = 7) | | | | |
|  | **Mean±SD** | **95% CI** | **p value** | **p value** | **Post** |  | **Mean±SD** | **95% CI** | **p value** | **p value** | **Post** |
|  |  |  | **Pre-Intermediate** | **Pre-Post** | **ES** |  |  |  | **Pre-Intermediate** | **Pre-Post** | **ES** |
| ***7^th^ session*** | | | | | | | | | | | |
| SBP rest (mmHg) | 109.3±10.2 | 99.9 - 118.7 | 0.052 | 0.021 | 2.03 |  | 117.1±7.6 | 110.2 -124.1 | 0.067 | 0.258 | 1.51 |
| SBP Intermediate (mmHg) | 122.9±9.5 | 114.1 - 131.7 |  |  |  |  | 130.0±12.9 | 118.1 - 141.9 |  |  |  |
| SBP post-exercise (mmHg) | 130.0±16.3 | 114.9 - 145.1 |  |  |  |  | 128.6±13.5 | 116.1 - 141.0 |  |  |  |
| DBP rest (mmHg) | 73.6±8.5 | 65.7 - 81.5 | 0.018 | 0.042 | 1.26 |  | 81.4±10.7 | 71.5 - 91.3 | 1 000 | 0.837 | -0.26 |
| DBP Intermediate (mmHg) | 82.9±7.6 | 75.9 - 89.8 |  |  |  |  | 81.4±9.0 | 73.1 - 89.8 |  |  |  |
| DBP post-exercise (mmHg) | 84.3^†^±9.8 | 75.3 - 93.3 |  |  |  |  | 78.6±9.0 | 70.25 - 86.89 |  |  |  |
| MBP rest (mmHg) | 85.5±8.7 | 77.4 - 93.5 | 0.022 | 0.019 | 1.61 |  | 93.3±8.6 | 85.4 - 101.3 | 0.522 | 0.960 | 0.22 |
| MBP Intermediate (mmHg) | 96.2±7.8 | 88.9 - 103.4 |  |  |  |  | 97.6±8.7 | 89.5 - 105.7 |  |  |  |
| MBP post-exercise (mmHg) | 99.5±10.4 | 89.9 - 109.2 |  |  |  |  | 95.2±8.4 | 87.5 - 102.9 |  |  |  |
| HR rest (bpm) | 78.3±11.4 | 67.8 - 88.8 | 0.001 | 0.002 | 3.32 |  | 85.4±16.6 | 70.1 - 100.7 | 0.001 | 0.001 | 2.34 |
| HR Intermediate (bpm) | 120.9±24.4 | 98.4 - 143.5 |  |  |  |  | 136.1±18.7 | 118.8 - 153.5 |  |  |  |
| HR post-exercise (bpm) | 116.2±21.6 | 96.3 - 136.1 |  |  |  |  | 124.3±19.7 | 106.1 - 142.6 |  |  |  |
| DP rest (bpm.mmHg) | 8625.0±1981.0 | 6792.3 - 10457.7 | 0.001 | 0.001 | 3.31 |  | 10078.6±2391.4 | 7866.9 - 12290.2 | 0.001 | 0.001 | 2.43 |
| DP Intermediate (bpm.mmHg) | 14879.5±3283.5 | 11842.8 - 17916.2 |  |  |  |  | 17816.7±3586.4 | 14499.8 -21133.5 |  |  |  |
| DP post-exercise (bpm.mmHg) | 15180.0±3607.0 | 11844.1 - 18515.9 |  |  |  |  | 15894.3±2206.9 | 13853.2 - 17935.4 |  |  |  |
| ***22^nd^ session*** | | | | | | | | | | | |
| SBP rest | 112.9±15.0 | 99.0 - 126.7 | 0.331 | 0.435 | 0.47 |  | 115.7±5.4 | 110.8 - 120.7 | 0.033 | 0.042 | 2.65 |
| SBP Intermediate | 121.4±13.5 | 109.0 - 133.9 |  |  |  |  | 131.4±10.7 | 121.5 - 141.3 |  |  |  |
| SBP post-exercise | 120.0±12.9 | 108.1 - 131.9 |  |  |  |  | 130.0±14.1 | 116.9 - 143.1 |  |  |  |
| DBP rest | 75.0±10.4 | 65.4 - 84.6 | 0.069 | 0.033 | 0.76 |  | 75.7±7.9 | 68.4 - 83.0 | 0.680 | 0.931 | 0.18 |
| DBP Intermediate | 82.1±11.5 | 71.5 - 92.8 |  |  |  |  | 78.6±10.7 | 68.7 - 88.5 |  |  |  |
| DBP post-exercise | 82.9±8.1 | 75.4 - 90.3 |  |  |  |  | 77.1±9.5 | 68.3 - 85.9 |  |  |  |
| MBP rest | 87.6±11.7 | 76.8 - 98.4 | 0.100 | 0.053 | 0.65 |  | 89.0±6.0 | 83.5 - 94.6 | 0.129 | 0.171 | 0.97 |
| MBP Intermediate | 95.2±11.8 | 84.3 - 106.2 |  |  |  |  | 96.2±9.3 | 87.6 - 104.8 |  |  |  |
| MBP post-exercise | 95.2±9.2 | 86.7 - 103.7 |  |  |  |  | 94.8±7.9 | 87.5 - 102.1 |  |  |  |
| HR rest | 86.3±19.9 | 67.9 - 104.7 | 0.001 | 0.034 | 1.12 |  | 85.4±6.1 | 79.8 - 91.1 | 0.001 | 0.001 | 6.25 |
| HR Intermediate | 119.3±25.1 | 96.1 - 143.5 |  |  |  |  | 132.5±16.7 | 117.0 - 148.0 |  |  |  |
| HR post-exercise | 108.6±19.3 | 90.8 - 126.4 |  |  |  |  | 123.5±24.0 | 101.3 - 145.7 |  |  |  |
| DP rest | 9675.7±2207.8 | 7633.9 - 11717.6 | 0.001 | 0.019 | 1.50 |  | 9877.1±730.8 | 9201.3 - 10553.0 | 0.001 | 0.001 | 8.41 |
| DP Intermediate | 14360.5±2766.3 | 11802.0 - 16918.9 |  |  |  |  | 17331.4±1777.9 | 15687.2 - 18975.7 |  |  |  |
| DP post-exercise | 12990.5±2428.3 | 10744.7 - 15236.2 |  |  |  |  | 16026.7*±3439.0 | 12846.1 - 19207.2 |  |  |  |
| ***35^th^ session*** | | | | | | | | | | | |
| SBP rest | 114.3±11.3 | 103.8 - 124.8 | 0.094 | 0.190 | 0.88 |  | 114.3±5.3 | 109.3 - 119.2 | 0.026 | 0.004 | 4.04 |
| SBP Intermediate | 128.6±16.8 | 113.1 - 144.1 |  |  |  |  | 132.9±17.0 | 117.1 - 148.6 |  |  |  |
| SBP post-exercise | 124.3±9.8 | 115.3 - 133.3 |  |  |  |  | 135.7±12.7 | 123.9 - 147.5 |  |  |  |
| DBP rest | 75.7±12.7 | 63.9 - 87.59 | 0.072 | 0.492 | 0.34 |  | 81.4±10.7 | 71.5 - 91.3 | 0.401 | 1 000 | 0.00 |
| DBP Intermediate | 84.3±11.3 | 73.8 - 94.8 |  |  |  |  | 86.4±12.5 | 74.9 - 98.0 |  |  |  |
| DBP post-exercise | 80.0±10.0 | 70.8 - 89.2 |  |  |  |  | 81.4±13.5 | 69.0 - 93.9 |  |  |  |
| MBP rest | 88.5±12.2 | 77.3 - 99.8 | 0.030 | 0.281 | 0.52 |  | 92.4±8.1 | 84.9 - 99.9 | 0.050 | 0.188 | 0.88 |
| MBP Intermediate | 99.0±12.6 | 87.4 - 110.7 |  |  |  |  | 101.9±12.4 | 90.4 - 113.4 |  |  |  |
| MBP post-exercise | 94.8±8.3 | 87.1 - 102.5 |  |  |  |  | 99.5±12.4 | 88.1 - 111.0 |  |  |  |
| HR rest | 83.9±19.4 | 65.9 - 101.8 | 0.001 | 0.007 | 1.54 |  | 82.4±11.0 | 72.3 - 92.6 | 0.001 | 0.001 | 3.68 |
| HR Intermediate | 122.0±29.9 | 94.3 - 149.6 |  |  |  |  | 130.6±21.3 | 110.8 - 150.3 |  |  |  |
| HR post-exercise | 113.7±27.8 | 88.0 - 139.4 |  |  |  |  | 122.9±21.9 | 102.6 - 143.1 |  |  |  |
| DP rest | 9584.3±2444.1 | 7323.9 - 11844.7 | 0.001 | 0.002 | 1.86 |  | 9430.0±1426.2 | 8111.0 - 10749.0 | 0.001 | 0.001 | 5.07 |
| DP Intermediate | 15802.4±4865.0 | 11303.0 -20301.8 |  |  |  |  | 17447.1±4006.8 | 13741.4 - 21152.9 |  |  |  |
| DP post-exercise | 14122.4±3581.5 | 10810.1 - 17434.7 |  |  |  |  | 16665.2±3189.5 | 13715.4 - 19615.0 |  |  |  |
| ***Biochemical parameters*** | | | | | | | | | | | |
| CT (mg/dL) | 165.2±19.8 | 144.4 - 186.0 |  | 0.232 | 0.54 |  | 188.0±33.7 | 152.7 - 223.3 |  | 0.825 | 0.36 |
| CT post (mg/dL) | 154.6±25.0 | 123.6 - 185.6 |  |  |  |  | 200.3*±7.6 | 181.5 - 219.1 |  |  |  |
| HDL (mg/dL) | 50.9±16.5 | 33.6 - 68.2 |  | 0.148 | 0.42 |  | 41.8±13.1 | 28.1 - 55.6 |  | 0.844 | 0.31 |
| HDL post (mg/dL) | 57.9±18.9 | 34.5 - 81.4 |  |  |  |  | 37.8±5.9 | 23.2 - 52.3 |  |  |  |
| LDL (mg/dL) | 91.3±12.0 | 78.7 - 103.9 |  | 0.745 | 0.32 |  | 114.2±24.8 | 88.2 - 140.3 |  | 0.698 | 0.46 |
| LDL post (mg/dL) | 87.4±18.5 | 64.6 - 110.4 |  |  |  |  | 125.7*±18.5 | 79.7 -171.6 |  |  |  |
| TG (mg/dL) | 115.3±63.1 | 49.1 - 181.6 |  | 0.230 | 0.63 |  | 182.0±18.5 | 159.0 - 205.0 |  | 0.928 | 0.12 |
| TG post (mg/dL) | 75.4±28.2 | 40.4 - 110.4 |  |  |  |  | 184.3*±25.4 | 121.2 - 247.5 |  |  |  |
| ***Food intake*** | | | | | | | | | | | |
| Total energy intake (kcal) | 1721.4±589.1 | 783.9 - 2658.8 |  | 0.209 | 0.34 |  | 1836.8±345.7 | 1407.6 - 2266.0 |  | 0.097 | 0.73 |
| Total energy intake (kcal) post | 1519.1±337.2 | 982.6 - 2055.6 |  |  |  |  | 1582.9±133.9 | 1416.5 - 1749.2 |  |  |  |
| % Carbohydrates | 47.5±5.7 | 38.5 - 56.5 |  | 0.731 | 0.26 |  | 47.2±11.1 | 33.4 - 61.0 |  | 0.333 | 0.41 |
| % Carbohydrates post | 49.0±6.3 | 39.0 - 59.0 |  |  |  |  | 51.8±10.1 | 39.2 - 64.4 |  |  |  |
| % Lipids | 34.0±2.9 | 29.3 - 38.7 |  | 0.347 | 1.62 |  | 29.2±4.8 | 23.2 - 35.2 |  | 0.106 | 1.00 |
| % Lipíds post | 29.3±8.4 | r15.9 - 42.7 |  |  |  |  | 24.4±4.1 | 19.3 - 29.5 |  |  |  |
| % Protein | 17.3±3.4 | 11.8 - 22.7 |  | 0.468 | 0.73 |  | 22.0±8.4 | 11.5 - 32.5 |  | 0.799 | 0.09 |
| % Protein post | 19.8±3.6 | 14.0 - 25.5 |  |  |  |  | 22.8±7.2 | 13.9 - 31.7 |  |  |  |

^†^p <0.05: Statistically significant group-by-time interactions

^*^p <0.05: Statistically significant difference between groups at the same time (general linear models-GLM with SIDAK post hoc test adjusted for antihypertensive treatment)

*Note*: G_RTBFR_: group of resistance training with blood flow restriction; G_TRT_: group of traditional resistance training; SBP: systolic blood pressure; DBP: diastolic blood pressure; MBP: mean blood pressure; HR: heart rate; DP: double product; TC: total cholesterol; HDL: high-density lipoprotein cholesterol; LDL: low-density lipoprotein cholesterol; TG: triglycerides.

**Table S2** Load (weight[kg] × nº of reps × nº of series*) for the exercises performed in both groups of training.

| **Session**  **of**  **training** | **Group** |  | **Weight (kg)** | | | | **Nº of reps** | | | | | **Mean load** | | | | | **Total**  **load** |
| --- | --- | --- | --- | --- | --- | --- | --- | --- | --- | --- | --- | --- | --- | --- | --- | --- | --- |
|  |  |  | Arm extension  (triceps) | Arm curl  (biceps) | Unilateral leg curl  (hamstring) | Leg extension  (quadriceps) | Arm extension  (triceps) | Arm curl  (biceps) | Right leg curl  (hamstring) | Left leg curl  (hamstring) | Leg extension  (quadriceps) | Arm extension  (triceps) | Arm curl  (biceps) | Right leg curl  (hamstring) | Left leg curl  (hamstring) | Leg extension  (quadriceps) |  |
| 1st to 6th  (Adaption phase) | G_RTBFR_ | M | 23.6 | 20.0 | 16.4 | 27.9 | 10.0 | 10.0 | 10 | 10.0 | 10.0 | 235.7 | 200.0 | 164.3 | 164.3 | 278.6 | 1042.9 |
|  |  | SD | 8.0 | 5.8 | 6.3 | 11.1 | 0.0 | 0.0 | 0.0 | 0.0 | 0.0 | 80.2 | 57.7 | 62.7 | 62.7 | 111.3 | 346.9 |
|  | G_TRT_ | M | 20.7 | 20.7 | 17.1 | 29.3 | 10.0 | 10.0 | 10 | 10.0 | 10.0 | 207.1 | 207.1 | 171.4 | 171.4 | 292.9 | 1050.0 |
|  |  | SD | 6.7 | 7.3 | 6.4 | 13.7 | 0.0 | 0.0 | 0.0 | 0.0 | 0.0 | 67.3 | 73.2 | 63.6 | 63.6 | 136.7 | 388.4 |
| 7th to 21st  (Specific phase) | G_RTBFR_ | M | 62.8 | 40.0 | 50.0 | 50.3 | 62.8 | 40.0 | 50.0 | 50.3 | 50.3 | 916.9 | 496.9 | 522.2 | 526.2 | 754.7 | 3216.8 |
|  |  | SD | 11.6 | 6.7 | 12.9 | 12.5 | 11.6 | 6.7 | 12.9 | 12.5 | 12.5 | 488.0 | 223.4 | 240.5 | 243.1 | 317.6 | 1421.6 |
|  | G_TRT_ | M | 29.2 | 25.5 | 30.2 | 30.9 | 29.2 | 25.5 | 30.2 | 30.9 | 30.9 | 880.7 | 792.2 | 729.5 | 743.4 | 1146.5 | 4292.3 |
|  |  | SD | 5.2 | 8.6 | 14.6 | 11.8 | 5.2 | 8.6 | 14.6 | 11.8 | 11.8 | 285.3 | 301.2 | 506.2 | 449.7 | 593.0 | 1932.7 |
| 22nd to 36st  (Specific phase) | G_RTBFR_ | M | 17.9 | 16.4 | 14.3 | 23.6 | 58.7 | 38.0 | 41.1 | 41.4 | 41.4 | 1083.8 | 643.2 | 586.0 | 596.9 | 793.7 | 3703.5 |
|  |  | SD | 5.7 | 4.8 | 4.5 | 5.6 | 16.2 | 10.3 | 11.4 | 12.9 | 12.9 | 563.6 | 314.8 | 210.6 | 246.3 | 272.4 | 1385.0 |
|  | G_TRT_ | M | 34.7 | 33.6 | 27.9 | 53.6 | 25.8 | 23.1 | 26.2 | 28.8 | 28.8 | 859.2 | 780.1 | 735.1 | 811.5 | 1261.0 | 4446.9 |
|  |  | SD | 10.7 | 11.1 | 7.6 | 25.0 | 5.5 | 3.7 | 7.1 | 5.5 | 5.5 | 171.3 | 297.5 | 338.3 | 300.9 | 556.8 | 1286.0 |

***one in adaption phase and three in specific phase (for each exercise).

*Note*: G_RTBFR_: group of resistance training with blood flow restriction; G_TRT_: group of traditional resistance training; M: Mean; SD: standard deviation.
